# Pathological pericyte expansion and impaired endothelial cell-pericyte communication in endothelial Rbpj deficient brain arteriovenous malformation

**DOI:** 10.3389/fnhum.2022.974033

**Published:** 2022-09-06

**Authors:** Samantha Selhorst, Sera Nakisli, Shruthi Kandalai, Subhodip Adhicary, Corinne M. Nielsen

**Affiliations:** ^1^Department of Biological Sciences, Ohio University, Athens, OH, United States; ^2^Honors Tutorial College, Ohio University, Athens, OH, United States; ^3^Neuroscience Program, Ohio University, Athens, OH, United States; ^4^Translational Biomedical Sciences Program, Ohio University, Athens, OH, United States; ^5^Molecular and Cellular Biology Program, Ohio University, Athens, OH, United States

**Keywords:** brain, endothelial, Notch, pericyte, Rbpj, vascular, arteriovenous malformation (AVM)

## Abstract

Pericytes, like vascular smooth muscle cells, are perivascular cells closely associated with blood vessels throughout the body. Pericytes are necessary for vascular development and homeostasis, with particularly critical roles in the brain, where they are involved in regulating cerebral blood flow and establishing the blood-brain barrier. A role for pericytes during neurovascular disease pathogenesis is less clear—while some studies associate decreased pericyte coverage with select neurovascular diseases, others suggest increased pericyte infiltration in response to hypoxia or traumatic brain injury. Here, we used an endothelial loss-of-function Recombination signal binding protein for immunoglobulin kappa J region (Rbpj)/Notch mediated mouse model of brain arteriovenous malformation (AVM) to investigate effects on pericytes during neurovascular disease pathogenesis. We tested the hypothesis that pericyte expansion, via morphological changes, and Platelet-derived growth factor B/Platelet-derived growth factor receptor β (Pdgf-B/Pdgfrβ)-dependent endothelial cell-pericyte communication are affected, during the pathogenesis of Rbpj mediated brain AVM in mice. Our data show that pericyte coverage of vascular endothelium expanded pathologically, to maintain coverage of vascular abnormalities in brain and retina, following endothelial deletion of Rbpj. In Rbpj-mutant brain, pericyte expansion was likely attributed to cytoplasmic process extension and not to increased pericyte proliferation. Despite expanding overall area of vessel coverage, pericytes from Rbpj-mutant brains showed decreased expression of *Pdgfrβ*, *Neural (N)-cadherin*, and *cluster of differentiation* (*CD)146*, as compared to controls, which likely affected Pdgf-B/Pdgfrβ-dependent communication and appositional associations between endothelial cells and pericytes in Rbpj-mutant brain microvessels. By contrast, and perhaps by compensatory mechanism, endothelial cells showed increased expression of *N-cadherin*. Our data identify cellular and molecular effects on brain pericytes, following endothelial deletion of Rbpj, and suggest pericytes as potential therapeutic targets for Rbpj/Notch related brain AVM.

## Introduction

Brain arteriovenous malformation (AVM) is a neurovascular disease characterized by multiple vascular abnormalities, including arteriovenous (AV) shunting—formation of direct connections between arteries and veins at the expense of capillary networks. Blood flows rapidly through these abnormal connections; thus, AVM vessels are prone to rupture, which can lead to devastating outcomes (Do Prado et al., 2019). Treatment methods for brain AVM are limited (Raper et al., 2020); therefore, it is important to understand the mechanisms of disease pathogenesis in order to develop novel therapies. Molecular and genetic studies from human tissue and animal models have revealed several signaling pathways involved in brain AVM pathogenesis, namely TGFβ (Bourdeau et al., 1999; Satomi et al., 2003; Park et al., 2009), Ras/Raf/MEK/ERK (Nikolaev et al., 2018; Fish et al., 2020), and Notch (Murphy et al., 2014; Nielsen et al., 2014). Collectively, these data support the emerging view that diverse mechanisms underlie brain AVM pathologies and diverse therapies must be developed to treat brain AVM patients safely and effectively.

Endothelial Notch signaling plays an essential role in vessel remodeling, angiogenesis, and endothelial cell specification in many vascular beds, including those in the central nervous system (CNS) (Krebs et al., 2000; Fernández-Chacón et al., 2021). We previously developed a mouse model of brain AVM, by selectively deleting Rbpj—a transcriptional regulator of canonical Notch signaling—from early postnatal endothelium in mice. Mutant mice developed AV shunts, displayed abnormal endothelial cell (EC) gene expression (suggesting disruption of arterial vs. venous EC identity), and showed altered smooth muscle cell coverage on the abnormal AV connections. In this mouse model, advanced brain AVM pathologies and 50% lethality were reported just 2 weeks post EC-Rbpj deletion (Nielsen et al., 2014); thus, endothelial Rbpj is required to prevent brain AVM formation in the early postnatal brain vasculature. Interestingly, careful regulation of Notch signaling is critical, as both loss and gain of function mutations in Notch signaling molecules result in abnormal vasculature in mice (Krebs et al., 2004; Murphy et al., 2014; Nielsen et al., 2014; Cuervo et al., 2016).

Pericytes are specialized perivascular cells closely associated with microvessels throughout the body. Pericytes and ECs are separated by a shared basement membrane, except at points of closer apposition called peg-and-socket contacts, which are typically found at the pericyte cytosolic processes that enwrap microvessels. Pericytes vary in morphology, function, and molecular signature, depending on where and when they are present (Uemura et al., 2020). Pericytes are necessary for vascular development and homeostasis and are particularly critical to brain capillaries for regulating cerebral blood flow (Bell et al., 2010, 2020; Gonzales et al., 2020) and establishing the blood-brain barrier (BBB) (Armulik et al., 2010; Daneman et al., 2010). Such developmental and functional influences on microvessels rely on intercellular signaling events between ECs and pericytes, permitting a “molecular crosstalk” that regulates vascular homeostasis.

Notch signaling is involved in EC-pericyte communication and association, by influencing pericyte maturation and recruitment to vessels (Liu et al., 2010; Gu et al., 2012). Notch achieves this, in part, by regulating Pdgf-B-Pdgfrβ signaling (Jin et al., 2008; Yao et al., 2011). In vasculature, Pdgf-B ligands are expressed and secreted by ECs (Battegay et al., 1994), while their membrane-localized Pdgf receptors are expressed primarily by pericytes (Winkler et al., 2010). In the CNS, impaired Pdgf-B-Pdgfrβ signaling leads to reduced pericyte coverage, vessel instability, and compromised BBB function (Armulik et al., 2010; Daneman et al., 2010; Nikolakopoulou et al., 2017). N-cadherin, another molecule involved in EC-pericyte interaction, is a membrane-spanning protein expressed on both ECs and pericytes (Gerhardt et al., 2000). N-cadherin mediates EC-pericyte communication, downstream of Pdgf-B-Pdgfrβ signaling, via direct contact between neighboring cells, and one study has implicated Notch and TGFβ as co-activators of N-cadherin (Li et al., 2011). Thus, Notch signaling promotes pericyte recruitment and adhesion to microvessels during normal neurovascular morphogenesis.

While pericytes are important for development and maintenance of the neurovascular system, a role for pericytes during neurovascular disease pathogenesis is less clear. Pericyte response to neurovascular disease (e.g., cerebrovascular and neurodegenerative diseases) varies, and pericytes adapt to their environment by changing morphology, undergoing differentiation, secreting growth factors, and altering vessel permeability to allow select solutes to reach affected brain tissue (Hirunpattarasilp et al., 2019; Lendahl et al., 2019). While several studies associate decreased pericyte coverage with neurovascular diseases like brain AVM (Chen et al., 2013; Tual-Chalot et al., 2014; Crist et al., 2018; Winkler et al., 2018; Diéguez-Hurtado et al., 2019; Nadeem et al., 2020), others demonstrate pericyte hypertrophy, tissue infiltration, and proliferation in response to hypoxia, ischemic stroke, or traumatic brain injury (Tagami et al., 1990; Bonkowski et al., 2011; Cai et al., 2017; Birbrair, 2019). Despite disparate consequences to pericytes during CNS diseases, these important vascular cells are indeed affected and may play actively pathogenic roles; thus, targeting pericytes in neurovascular anomalies, like brain AVM, is a promising therapeutic avenue (Lebrin et al., 2010; Thalgott et al., 2015; Geranmayeh et al., 2019).

We investigated consequences to pericytes in our endothelial Rbpj mediated model of brain AVM, and we found that CNS pericytes pathologically expanded in a regionally and temporally regulated manner, as compared to controls. In Rbpj mutant cerebellum and cortex (but not brain stem), increased pericyte area kept pace with increased endothelial area, thus maintaining pericyte coverage of microvessels. Pathological pericyte expansion increased in severity as features of brain AVM (AV shunting) progressed; however, there was no evidence for early pericyte reduction, indicating temporal regulation of brain pericytes by endothelial Rbpj. Pericyte expansion was not caused by increased cell proliferation, but rather by hypertrophy of cytosolic pericyte processes on enlarging AV connections. Rbpj dependent EC-pericyte communication was affected in mutant brain vasculature, as isolated brain pericytes showed decreased expression of *Pdgfrβ*, *N-cadherin*, and *CD146*—all of which are molecules involved in pericyte recruitment to and association with microvessels. By contrast, isolated brain ECs showed increased expression of *N-cadherin*, perhaps in response to downregulation by pericytes. Our results indicate that pathological pericyte expansion progresses in concert with AV shunt formation, during Rbpj mediated brain AVM; thus, our data challenge a working hypothesis in the field, which posits that decreased pericyte coverage necessarily precedes brain AVM formation. Collectively, our findings define a novel role for Rbpj in postnatal brain endothelium—to prevent pathological pericyte expansion and to maintain pericyte morphology. Our study also refines the current view of pericyte involvement in neurovascular disease and the therapeutic potential of targeting and manipulating pericytes in brain AVM.

## Materials and methods

### Mice

All experiments were completed in accordance with Ohio University’s Institutional Animal Care and Use Committee (IACUC) protocol number 16-H-024. Mouse lines *Cdh5(PAC)-CreER*^T2^** (Sörensen et al., 2009), *Rbpj^flox^* (Tanigaki et al., 2002), *Rosa26*^mT/mG^** (Muzumdar et al., 2007), and *Cspg4(NG2)-DsRed* (Zhu et al., 2008) were, respectively, provided by Taconic Biosciences (in accord with Breeding Agreement), Tasuku Honjo (Kyoto University), and the last two by Jackson Laboratory [*GT(Rosa)26Sor*^*TM*4(*ACTB*–*tdTomato–EGFP)Luo*^: JAX stock #007576 and *Tg(Cspg4-DsRed.T1)1Akik:* JAX stock #008241]. At P1 and P2, 100 μg of Tamoxifen (Sigma) in 50 μL of peanut oil (Planters) was injected intragastrically, as previously described (Nielsen et al., 2014). PCR based genotyping was performed, as previously described (Nielsen et al., 2014), except *Rosa26*^mT/mG^** and *Cspg4(NG2)-DsRed* genotyping was determined by tail biopsy tissue fluorescence, using a Nikon NiU microscope.

### Tissue harvest and preparation

Brain and retina tissue was harvested following intracardial perfusion and simultaneous euthanasia by exsanguination. Perfusion solution depended on subsequent analysis, as follows: 1% paraformaldehyde (PFA) for anti-CD13, anti-N-cadherin, anti-Pdgfrβ, NG2-DsRed; phosphate buffered saline (PBS) for anti-desmin. Endothelial cells were genetically labeled with mGFP or were perfusion labeled with intravenous Dylight488 *Lycopersicon esculentum* (tomato) lectin (Vector Laboratories) (50 μg lectin/125–150 μL PBS). For tissue sections, brains were hemisected, cryopreserved in 30% sucrose, and stored at −80°C. Mid-sagittal cryosections, 10–12 μm thick, were collected with a CM-1350 cryostat (Leica) and stored at −80°C. For mid-sagittal sections, the following brain regions were imaged: (i) frontal cortex near the pial surface was imaged; (ii) cerebellum lobule V, VIA, VIB, or VII was imaged, as these lobules are consistently affected in Rbpj^*i*ΔEC^ mutants (Chapman et al., 2022); (iii) brain stem immediately caudal to the pons (as identified by the pontine flexure) was imaged. For whole cortex preparation, a 2–3 mm thick slice of frontal cortex was removed with a scalpel. For whole retina preparation, retinae were dissected and splayed, according to published methods (Tual-Chalot et al., 2013).

### Immunostaining and 5-ethynyl-2′-deoxyuridine incorporation

Immunostaining was performed with modifications from standard protocol previously described (Nielsen and Dymecki, 2010). Primary antibody dilutions: rat anti-CD13 (MCA2183GA) (1:500) (AbD Serotec); mouse anti-desmin (D33) (1:50) (DAKO/Agilent); mouse anti-N-cadherin (3B9) (1:300) (Invitrogen); rat anti-Pdgfrβ (CD140b) (1:50) (Invitrogen). Secondary antibodies: Cy3 donkey anti-rat; Alexa647 donkey anti-mouse; Alexa647 donkey anti-rat (all dilutions 1:500) (Jackson ImmunoResearch). For EdU incorporation assay, mice were injected intraperitoneally with 10 μg/gram of body weight with EdU at P5–7 or P8–10 or P12–14. On the day of harvest (P7 or P10 or P14), tissue was harvested 2 h post-injection. Detection of EdU used Click-iT™ Plus chemistry, with AlexaFluor^®^ 647 component following manufacturer’s instructions (Invitrogen). Cell nuclei were counterstained with 4′,6′-diamidino-2-phenylindole (DAPI). Tissue sections were mounted with ProLong Gold™ (Invitrogen) and coverslipped for imaging.

### Single cell suspension and cell isolation

Whole brain tissue was mechanically and chemically dissociated by Neutral Protease (Worthington), Collagenase Type II (Worthington), Deoxyribonuclease (Worthington), and Complete 1X DMEM (Gibco) with 5% heat inactivated Fetal Bovine Serum (Gibco) and 1% PenStrep (Gibco). 70% Percoll (Cytiva) density gradient was used to separate single cell suspension into cellular fractions. Endothelial cells were labeled with CD31 microbeads (1:10) (mouse, Miltenyi Biotec). Pericytes were labeled with primary antibody rat anti-mouse CD13 (1:50) (Bio-Rad) and secondary anti-rat IgG MicroBeads (1:10) (Miltenyi Biotec). Select cell populations were isolated *via* magnetic activated cell sorting using LS separation columns (QuadroMACS™ Starting Kit, Miltenyi Biotec). To reduce endothelial cell contamination in the pericyte samples, endothelial cells were removed prior to collecting pericytes. Cell pellets were stored at −80°C until RNA extraction.

### Reverse transcription quantitative PCR

Total RNA was extracted from isolated cells using RNeasy Plus Micro Kit (including genomic DNA removal columns) (Qiagen), and concentration was measured using NanoDrop One spectrophotometer. For Reverse transcription quantitative PCR (RT-Qpcr), RNA input was 10 ng per well (96-well plate format). qScript One-Step SYBR Green RT-qPCR (Quantabio) was used to run qPCR with cycling in a CFX Connect Real-Time PCR Detection System (BioRad), using CFX Maestro 2.0 Software for Windows PC (BioRad). Tissue from 3 to 5 brains was pooled for each biological sample. Each biological sample was run in technical triplicate. Cycling conditions were as follows: (1) 49°C 10 min; (2) 95°C 5 min; (3) 95°C 10 s; (4) 58°C 30 s; (5) repeat steps 3–4 39 times; (6) melt curve 55–95°C 5 s, in 0.5°C increments. Quantification was performed using the comparative *C*_*T*_ method with Microsoft Excel software (Schmittgen and Livak, 2008). Values were normalized to expression of reference genes *β-actin* for ECs and *Rplpo* for pericytes. Reference genes were selected based on test runs for three reference genes (*β-actin*, *Gapdh*, *Rplpo*) per cell type—genes with consistent results across triplicate runs were selected. All sample replicates were used in quantification analyses (outliers were not identified; no samples were removed from analysis). Primer sequences and information relevant to MIQE Guidelines are listed in Supplementary Table 1.

### Western blotting

Protein lysates were prepared in 1X RIPA buffer from isolated cells (see above). Protein concentration was estimated using Precision Red (Cytoskeleton, Inc.) and NanoDrop One spectrophotometer. Proteins were separated using an 8% polyacrylamide separating/resolving gel, 4% stacking gel, and electrophoresis. Protein was transferred to PVDF membrane, and membrane was blocked with 3% bovine serum albumin in tris-buffered saline with Tween-20, before incubating with primary antibodies. Primary antibody dilutions: rabbit anti-gapdh (1:1,000) (Cell Signaling Technologies); mouse anti-N-cadherin (3B9) (1:1,000) (Invitrogen); rat anti-Pdgfrβ (CD140b) (1:1,000) (Invitrogen); rabbit anti-Rbpj (1:1,000) (Cell Signaling Technologies). HRP-conjugated secondary antibody dilutions: anti-mouse (1:1,000); anti-rabbit (1:1,000); anti-rat (1:1,000) (Cell Signaling Technologies). Chemiluminescent substate was applied to the membrane and bands were detected and quantified with BioRad ChemiDoc XRS+ and ImageLab software.

### Fluorescence imaging

Epifluorescent images were acquired using a Nikon NiU microscope and NIS Elements software. Confocal fluorescent images were acquired using a Zeiss LSM 510 laser scanning microscope system and Upright Zen software. Confocal Z-stacks ranged from 36 to 40 slices, at 2 μm steps, and were projected at maximum intensity.

### Quantification and statistical analysis

For pericyte ensheathment ratio, following retina imaging, a 10 by 10 grid was overlayed with the mGFP*+* endothelial cell images and desmin+ or CD13+ pericyte images in Adobe Photoshop (Creative Cloud). Points of intersection between the grid and positive cells were counted, modified from previously published protocols (Chan-Ling et al., 2004; Hughes et al., 2007), as a proxy for measuring changes to cell area or direct pericyte coverage of endothelium. Statistical analysis was completed using Prism software (GraphPad). Except for qPCR data (see above), unpaired Student’s *t*-tests with Welch’s correction were used to compare values between control and mutant mice. *P*-values < 0.05 were considered significant (*P < 0.05, ^**^P < 0.01, ^***^P < 0.001, ^****^P < 0.0001, ns = not significant).

## Results

### Central nervous system pericyte area was pathologically expanded by P10 in cerebellum and by P14 in frontal cortex and retina, following endothelial deletion of Rbpj from birth

**FIGURE 1:**
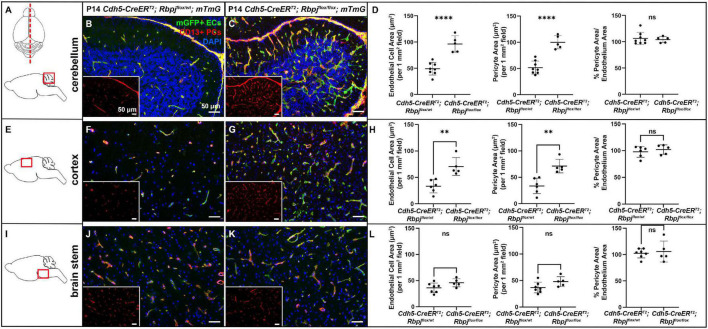
CD13-positive cortex and cerebellum pericyte area expanded and kept pace with expanded endothelium at P14, following endothelial deletion of Rbpj. In all tissue panels, CD13+ pericytes (PCs) (red), mGFP+ ECs (green), DAPI+ nuclei (blue); insets show CD13+ pericytes only. **(A)** Upper schematic indicates mid-sagittal plane of section through brain. Lower schematic indicates cerebellum region shown. **(B,C)** mGFP+ endothelial area and CD13+ pericyte area pathologically expanded in P14 Rbpj^iΔEC^ cerebellum, as compared to controls. Quantified in **(D)** endothelial area *P* < 0.0001; pericyte area *P* < 0.0001. Percentage of pericyte area/endothelial area did not change (right graph in **D**; *P* = 0.7048. *N* = 8 controls and *N* = 5 mutants. **(E)** Schematic indicates cortex region shown. **(F,G)** mGFP+ endothelial area and CD13+ pericyte area pathologically expanded in P14 Rbpj^iΔEC^ cortex, as compared to controls. Quantified in **(H)** endothelial area *P* = 0.0022; pericyte area *P* = 0.0016. Percentage of pericyte area/endothelial area did not change (right graph in **H**; *P* = 0.5015). *N* = 6 controls and *N* = 5 mutants. **(I)** Schematic indicates brainstem region shown. **(J,K)** mGFP+ endothelial area and CD13+ pericyte area did not change in P14 Rbpj^iΔEC^ brain stem, as compared to controls. Quantified in **(L)** endothelial area *P* = 0.0835; pericyte area *P* = 0.0799. Percentage of pericyte area/endothelial area did not change (right graph in **L**; *P* = 0.6973). *N* = 7 controls and *N* = 5 mutants. ^**^P < 0.01; ^****^P < 0.0001; ns, not significant.

To delete Rbpj from ECs in a temporally restricted manner, we bred *Cdh5(PAC)-CreER*^T2^*; Rbpj*^flox/wt^** and *Cdh5(PAC)-CreER*^T2^*; Rbpj*^flox/flox^**, hereafter referred to as control and Rbpj^iΔEC^ mutant mice, and we administered Tamoxifen to induce endothelial Rbpj deletion at postnatal day (P) 1 and P2 (Nielsen et al., 2014). Effective deletion of Rbpj from ECs was confirmed by P7 in brain ECs isolated from Rbpj^iΔEC^ mice, as compared to controls (Supplementary Figure 1). This corroborated published data that showed loss of Rbpj protein from cortical ECs and cerebellum ECs (Nielsen et al., 2014; Chapman et al., 2022) in P14 Rbpj^iΔEC^ brain tissue, as compared to controls. To determine whether total pericyte bed area, within mid-sagittal section through brain tissue, was altered in Rbpj^iΔEC^ mice, we immunostained against the pericyte marker CD13 and measured CD13+ area per tissue area. To determine total endothelial area, we genetically labeled ECs with mGFP from the Cre-responsive *Rosa26*^mT/mG^** (*mTmG*) allele (Nielsen et al., 2014) and measured mGFP+ area per tissue area. By P14, CD13+ pericyte area and mGFP+ endothelial area (per brain tissue area) increased in Rbpj^iΔEC^ mutant cerebellum (Figures 1A–D) and cortex (Figures 1E–H), but not in brain stem (Figures 1I–L), as compared to controls [note that our endothelial area results were similar to previously published results that showed increased vascular density in P14 Rbpj^iΔEC^ mutants, as compared to controls (Nielsen et al., 2014)]. Interestingly, the percentage of pericyte area per endothelial area was not changed in mutant vs. control (right graphs in Figures 1D,H,L), suggesting that overall pericyte coverage of brain vessels did not change. Rather, pericyte expansion kept pace with pathological endothelial expansion. To illustrate pericyte-microvessel congruency and coverage of microvessels, we acquired higher magnification images of CD13+ pericytes and mGFP+ microvessels in P14 control and mutant cerebellum (Supplementary Figures 2B,C), cortex (Supplementary Figures 2D,E), and brain stem (Supplementary Figures 2F,G). Because no pan-pericyte marker has been identified to date, we repeated our pericyte-positive area measurements using tissue immunostained against the pericyte marker desmin. Using desmin+ pericytes and Dylight488-lectin+ ECs for area measurements, we found similarly increased pericyte and endothelial expansion in P14 Rbpj^iΔEC^ mutant cerebellum (Supplementary Figures 3A–D) and frontal cortex (Supplementary Figures 3E–H), but not brain stem (Supplementary Figures 3I–L), as compared to controls. Percentage pericyte/endothelial area was not changed in any brain region, indicating desmin+ pericyte expansion kept pace with endothelial expansion (right graphs in Supplementary Figures 3D,H,L). These data suggest that endothelial Rbpj is required, in different brain regions, to prevent pericytes from expanding along with expanding endothelium in the early postnatal brain.

Because brain AVM has been correlated with pericyte reduction in other studies, and to examine pericyte coverage at different timepoints post-endothelial Rbpj deletion, we analyzed pericyte area from pre-brain AVM (P7) and from earlier (P10)- and later (P21)-stage brain AVM. While the precise onset of Rbpj^iΔEC^ brain AVM in mice has not been reported, previous work showed that increased AV connection (microvessel) diameter appeared around P8–P10 (Nielsen et al., 2014). Thus, we decided to analyze brain pericyte area at P7, a timepoint before features of brain AVM developed. In P7 Rbpj^iΔEC^ mutants, as compared to controls, endothelial area was not increased (Supplementary Figures 4A–C,E–G, quantified in left graphs in D,H), suggesting that endothelial expansion, a feature of brain AVM onset, had not yet occurred. At P7, CD13+ pericytes were observed in cerebellum and cortex of controls and Rbpj^iΔEC^ mutants (Supplementary Figures 4A–C,E–G, middle graphs in D,H), and pericyte coverage of microvessels was similar in controls and Rbpj^iΔEC^ mutants (Supplementary Figure 4, right graphs in D,H). These data indicate that brain pericytes were present, prior to the onset of brain AV features, and suggest that pericyte reduction is likely not necessary for Rbpj^iΔEC^ brain AVM to form. In P10 Rbpj^iΔEC^ mutants, as compared to controls, pericytes were present in all brain regions analyzed; however, increased CD13+ pericyte area and mGFP+ endothelial area was only seen in cerebellum, with pericyte expansion in pace with endothelial expansion (Supplementary Figures 5A–D). Increased CD13+ pericyte area and mGFP+ endothelial area was not observed in P10 Rbpj^iΔEC^ mutant cortex (Supplementary Figures 5E–H) or brain stem (Supplementary Figures 5I–L), as compared to controls. Using desmin as a marker for P10 pericytes, we did not measure any changes in desmin+ pericyte area in any brain region from Rbpj^iΔEC^ mutant vs. control (Supplementary Figures 6A–L). To determine whether pericytes were still present (and pathologically expanded) on vessels after advanced AV shunts have formed, we next analyzed P21 brain tissue. We found increased mGFP+ endothelial area and CD13+ pericyte area in Rbpj^iΔEC^ cerebellum (Supplementary Figures 7A–D) and cortex (Supplementary Figures 7E–H), but not in brain stem (Supplementary Figures 7I–L), as compared to controls. Pericyte area expanded in concert with endothelial area, as the percentage of pericyte area/endothelial area did not change in Rbpj^iΔEC^ vs. control brain tissue (right graph in Supplementary Figure 7L). A summary of Rbpj^iΔEC^ brain pericyte expansion, over time and in select brain regions, is included in Table 1. These data, coupled with the previous finding that only 50% of Rbpj^iΔEC^ mice survive to P14 (Nielsen et al., 2014), highlighted P14 as a timepoint for further analyses. Together, our findings suggest a temporal requirement for endothelial Rbpj to prevent pathological pericyte expansion in the early postnatal brain.

**TABLE 1:** Summary of regional and temporal pericyte expansion (as measured by different pericyte markers) in the CNS vasculature, following endothelial deletion of Rbpj.

Central nervous system region		Degree of postnatal (P) pericyte expansion in Rbpj^iΔEC^ vs. control		
		P10	P14	P21
	**Cerebellum**	↑ CD13	↑↑↑↑ CD13	↑↑ CD13
		no desmin	↑↑ desmin	
	**Cortex (frontal)**	no CD13	↑↑ CD13	↑ CD13
		no desmin	↑↑ desmin	
	**Brain stem**	no CD13	no CD13	no CD13
		no desmin	no desmin	
	**Retina**	no CD13	↑ CD13	
		↑↑↑ desmin	↑↑↑ desmin	

↑ represents *(P < 0.05) statistical significance.

↑↑ represents **(P < 0.01) statistical significance.

↑↑↑ represents ***(P < 0.001) statistical significance.

↑↑↑↑ represents ****(P < 0.0001) statistical significance.

The mouse retina is often used in vascular studies as part of the CNS and as a proxy for studies in the brain. While retinal AVM have not been reported in endothelial Rbpj mutants, previous studies showed that loss of Notch signaling molecules from ECs leads to increased angiogenic sprouting and branch points in the leading edge of early postnatal retinal vasculature (Hellström et al., 2007). Given our results from brain vasculature, we hypothesized that endothelial Rbpj may also be required in the neonatal retinal vasculature to prevent pericyte expansion. We dissected eyes from P10 to P14 control and Rbpj^iΔEC^ mice and used a whole mount preparation to splay the eye and expose the retina for immunostaining and tissue mounting (Supplementary Figure 8A). We labeled pericytes with CD13 and desmin markers, and because we stained whole tissue, we measured a pericyte ensheathment ratio (PER) as a proxy for pericyte+ and endothelium+ area measurements. Our PER method was adapted from previous studies with retinal ECs and pericytes (Chan-Ling et al., 2004; Hughes et al., 2007). We overlayed a 10x10 grid onto a retina image (Supplementary Figure 8B) and counted the number of intersection points at which pericyte+ or EC+ marker was observed (Supplementary Figures 8C,D,F,G). At P10, we did not observe increased number of EC+ intersection points in control vs. Rbpj^iΔEC^ (left graphs in Supplementary Figures 8E,H), in agreement with previous data suggesting that endothelium had not yet expanded in Rbpj^iΔEC^ brain. At P10, we observed increased number of desmin+ pericyte intersection points but not CD13+ points (middle graphs in Supplementary Figures 8E,H). Consistent with data from brain vessels, the ratio of pericyte+/EC+ intersection points in Rbpj^iΔEC^ vs. controls was not affected by P10 (right graphs in Supplementary Figures 8E,H). In P14 retina, the number of EC+ and pericyte+ intersection points significantly increased in Rbpj^iΔEC^, as compared to controls, using either CD13 (Supplementary Figures 8I–K) or desmin (Supplementary Figures 8L–N) to label pericytes. However, the ratio of pericyte+/EC+ intersection points in Rbpj^iΔEC^ vs. controls was not affected (right graphs in Supplementary Figures 8K,N). A summary of Rbpj^iΔEC^ retinal pericyte expansion, over time, is included in Table 1. These data suggest that pericyte expansion kept pace with endothelial expansion in the early postnatal retinae, following endothelial deletion of Rbpj.

### The number of pericytes per brain vessel length increased by P14, without increased cell proliferation, following endothelial deletion of Rbpj from birth

**FIGURE 2:**
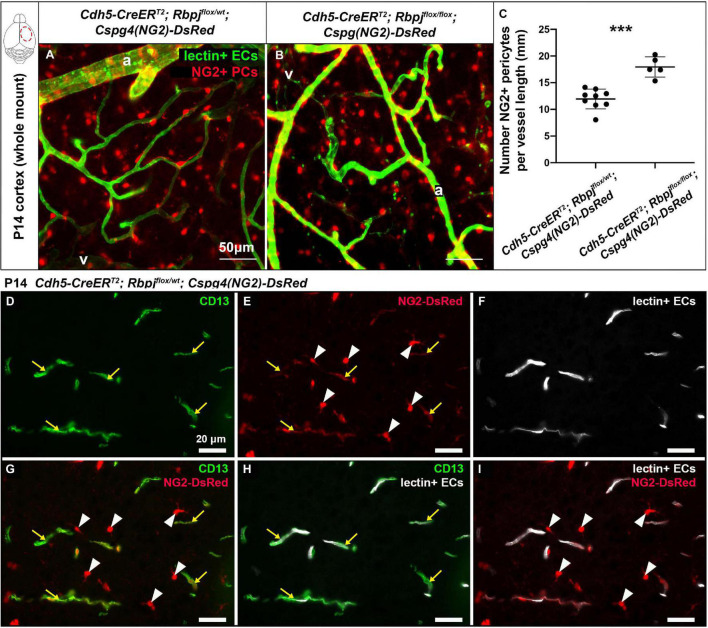
Endothelial Rbpj deficiency led to increased number of NG2-positive pericytes per vessel length by P14. **(A,B)** In P14 whole mount cortex, pericytes were labeled by the *Cspg4(NG2)-DsRed* transgene (red). ECs were labeled by Dylight488-lectin (green). NG2+ pericytes (PCs) were counted, and the corresponding lectin+ vessel length [between arteries (a) and veins (v)] was measured. The number of NG2+ pericytes per millimeter of vessel length significantly increased in Rbpj^iΔEC^ mutants, as compared to controls (quantified in **C**; *P* = 0.0004, *N* = 9 controls and *N* = 5 mutants). **(D–I)** P14 *Cspg4(NG2)-DsRed* (red) mid-sagittal brain tissue sections were immunostained against CD13 (green), and vessels were highlighted with Dylight488-lectin (white). **(D)** CD13 labeled pericytes; **(E)** *Cspg4(NG2)-DsRed* genetically labeled pericytes (yellow arrows) and oligodendrocyte precursor cells (white arrowheads); **(F)** Perfused Dylight488-lectin labeled ECs within blood vessels. **(G)** Co-labeling showed CD13+/Cspg4(NG2)-DsRed+ pericytes (yellow arrows) and CD13-/Cspg4(NG2)-DsRed+ oligodendrocyte precursor cells (white arrowheads). **(H,I)** Pericytes were found adjacent to lectin+ vessel, while oligodendrocyte precursor cells were not found near vessels. ^***^P < 0.001.

To determine whether pericyte expansion resulted from an increased number of pericytes, we counted the number of pericytes, per vessel length, on AV connections (4–6 μm diameter capillaries in control and > 12 μm diameter AV shunts in Rbpj^iΔEC^ brains). We used another pericyte marker, the transgene *Cspg4(NG2)-DsRed* (Zhu et al., 2008), which does not label pericyte processes, so that individual pericytes could be readily distinguished. We bred the transgene into our P14 control and Rbpj^iΔEC^ mice and counted Cspg4(NG2)-DsRed+ pericytes per Dylight488-lectin+ vessel length (mm). Using a whole mount cortical preparation so that intact vessels and pericytes could be visualized, described in Nielsen et al. (2014), we found an increased number of pericytes per mm of vessel in Rbpj^iΔEC^ cortex at P14, as compared to controls (Figures 2A–C). Because *Cspg4(NG2)* is highly expressed by oligodendrocyte precursor cells (Polito and Reynolds, 2005; Zhu et al., 2008), we wanted to validate that the cells we identified as Cspg4(NG2)+ and directly juxtaposed to vessels were indeed pericytes. We immunostained Dylight488-lectin-injected, *Cspg4(NG2)-DsRed* brain tissue against CD13 in P14 controls. We found CD13+/Cspg4(NG2)-DsRed+ pericytes were identified adjacent to lectin+ microvessels (Figures 2D–I), while CD13-/Cspg4(NG2)-DsRed+ oligodendrocyte precursor cells were not found near vessels (Figures 2E,G,I).

**FIGURE 3:**
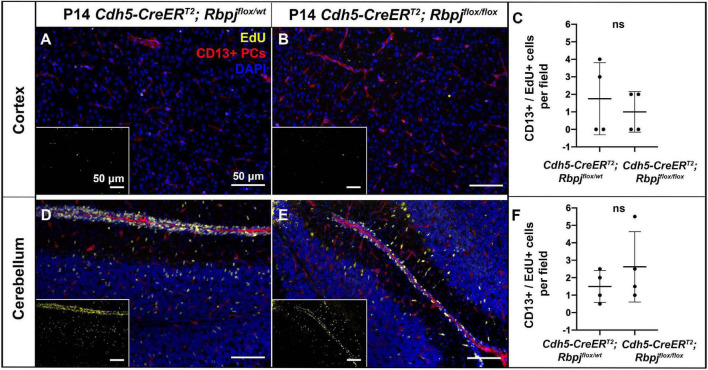
Endothelial deletion of Rbpj did not lead to increased CD13-positive pericyte proliferation from P12 to P14 in cortex or cerebellum. In all tissue panels, CD13+ pericytes (PCs) (red), EdU+ nuclei (yellow), DAPI+ nuclei (blue). **(A,B)** Mice were administered EdU at P12, P13, P14. Mid-sagittal sections through P14 control and Rbpj^iΔEC^ cortex. CD13+ pericytes and CD13+/EdU+ double positive pericytes were counted at P14 harvest. **(C)** Quantification of CD13+ pericytes per CD13+/EdU+ pericytes showed no significant change (*P* = 0.555). *N* = 4 controls and *N* = 4 mutants. **(D,E)** Mid-sagittal sections through P14 control and Rbpj^iΔEC^ cerebellum. CD13+ pericytes and CD13+/EdU+ double positive pericytes were counted at P14 harvest. **(F)** Quantification of CD13+ pericytes per CD13+/EdU+ pericytes showed no significant change (*P* = 0.364). *N* = 4 controls and *N* = 4 mutants. ns, not significant.

To assess pericyte proliferation during AV shunt formation, we initiated EdU incorporation experiments during incremental time periods before P14. We administered EdU on three consecutive days at P5–7, P8–10, P12–14, and harvested tissue on P7, P10, P14, 2 h post-EdU. We counted double-labeled EdU+ and CD13+ pericytes in mid-sagittal brain tissue sections. Overall, we found very few proliferative pericytes in control and mutant brain tissue. Our analysis did not detect a significant difference in the number of proliferative pericytes (per brain tissue field) between Rbpj^iΔEC^ and control mice, in cerebellum or cortex, and at all timepoints analyzed (P14 cortex, Figures 3A–C; P14 cerebellum Figures 3D–F) (P7 cortex, Supplementary Figures 9A–C; P7 cerebellum Supplementary Figures 9D–F; P10 cortex, Supplementary Figures 9G–I; P10 cerebellum Supplementary Figures 9J–L). Taken together, these results suggest that the increased number of pericytes per vessel length was not caused by increased cell proliferation but by another mechanism, perhaps related to another cellular change to pericytes and/or to changes in the Rbpj^iΔEC^ microvessels.

### Brain pericytes extended processes to enwrap late-stage arteriovenous shunts, following endothelial deletion of Rbpj

**FIGURE 4:**
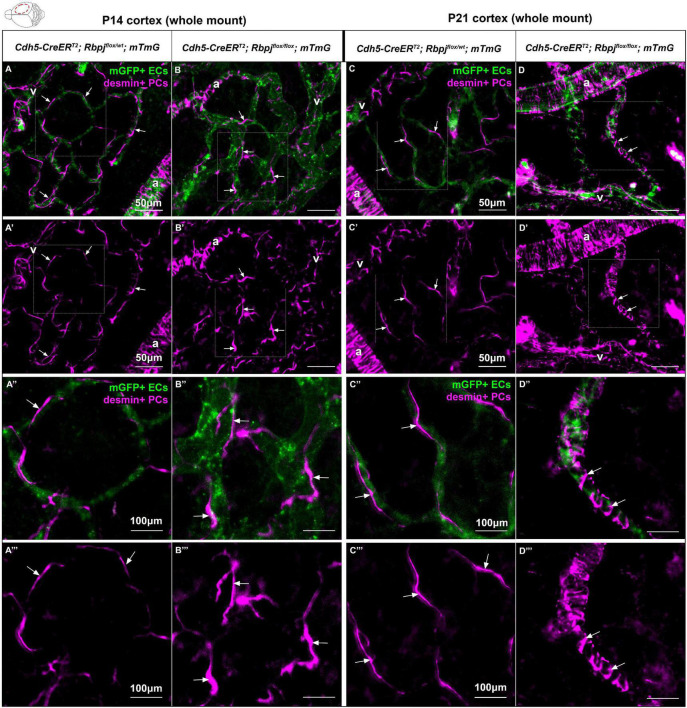
Desmin-positive pericytes showed abnormal morphology on P14 and P21 brain microvessels, following endothelial deletion of Rbpj. Whole mount immunostaining against desmin highlighted pericyte processes (PCs) (magenta) on mGFP+ brain AV connections (ECs) (green). By P14 **(A–B”’)** and P21 **(C–D”’)**, pericyte processes enwrapped brain capillaries in controls and AV shunts in Rbpj^iΔEC^ mutants (arrows). **(B’,B”’,D’,D”’)** Pericytes extended their cytosolic processes to maintain coverage of expanding endothelium. By P21, pericytes on AV shunts appeared to acquire a ring-like morphology to enwrap the expanded microvessel. A, artery; V, vein. P14, *N* = 9 controls and *N* = 9 mutants; P21, *N* = 3 controls and *N* = 3 mutants.

To determine whether pericyte morphology was affected on Rbpj^iΔEC^ brain AV connections, we combined our whole mount cortical prep with anti-desmin immunostaining. Desmin expression spanned the cytosolic pericyte processes and offered a view of desmin+ pericyte ensheathment on mGFP+ brain vessels. In P14 control cortex, pericytes on AV connections (4–6 μm diameter capillaries) extended thin cytoplasmic processes to enwrap vessels (Figures 4A–A”’), while in Rbpj^iΔEC^ cortex, pericyte processes appeared thickened and ring-like in abnormal AV vessel segments (>12 μm diameter AV shunts) (Figures 4B–B”’). In P21 control cortex, pericytes were similar to P14 controls, with thin and elongated processes (Figures 4C–C”’). In P21 Rbpj^iΔEC^ cortex, thickened, ring-like pericytes began to resemble the concentric morphology characteristic of vascular smooth muscle cells (Figures 4D–D”’). These findings suggest that endothelial Rbpj is required to maintain healthy pericyte morphology on brain microvessels.

### Endothelial Rbpj deficiency led to decreased Pdgfrβ expression in pericytes and decreased vessel coverage by Pdgfrβ+ pericytes

**FIGURE 5:**
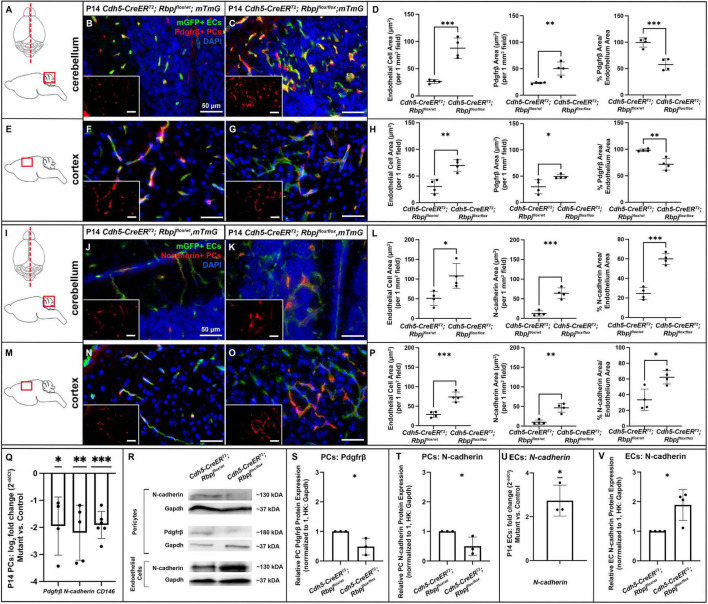
By P14, endothelial Rbpj deficiency led to decreased expression of *Pdgfr*β, *N-cadherin*, and *CD146* by brain pericytes and increased expression of *N-cadherin* by brain endothelial cells. **(A,I)** Upper, schematic of brain mid-sagittal section cerebellum; lower, schematic of cerebellum brain region shown. **(E,M)** schematic of cortex brain region shown. **(B,C,F,G)** Pdgfrβ+ pericytes (PCs) (red), mGFP+ ECs (green), DAPI+ nuclei (blue). **(B,C)** mGFP+ endothelial area and Pdgfrβ+ pericyte area increased in P14 Rbpj^iΔEC^ cerebellum, as compared to control. Quantified in **(D)** endothelial area *P* = 0.0007; Pdgfrβ+ area *P* = 0.0048. Percentage of Pdgfrβ+ area/endothelial area was decreased in Rbpj^iΔEC^ cerebellum, as compared to controls (right graph in **D**; *P* = 0.0010). *N* = 4 controls and *N* = 4 mutants. **(F,G)** mGFP+ endothelial area and Pdgfrβ+ pericyte area increased in P14 Rbpj^iΔEC^ cortex, as compared to control. Quantified in **(H)** endothelial area *P* = 0.0033; Pdgfrβ+ area *P* = 0.0317. Percentage of Pdgfrβ+ area/endothelial area was decreased in Rbpj^iΔEC^ cerebellum, as compared to controls (right graph in **H**; *P* = 0.0032). *N* = 4 controls and *N* = 4 mutants. **(J,K,N,O)** N-cadherin+ cells (red), mGFP+ ECs (green), DAPI+ nuclei (blue). **(J,K)** mGFP+ endothelial area and N-cadherin+ area increased in P14 Rbpj^iΔEC^ cerebellum, as compared to control. Quantified in **(L)** endothelial area *P* = 0.0204; N-cadherin+ area *P* = 0.0005. Percentage of N-cadherin+ area/endothelial area decreased in Rbpj^iΔEC^ cerebellum, as compared to controls (right graph in **L**; *P* = 0.0002). *N* = 4 controls and *N* = 4 mutants. **(N,O)** mGFP+ endothelial area and N-cadherin+ area increased in P14 Rbpj^iΔEC^ cortex, as compared to control. Quantified in **(P)** endothelial area *P* = 0.0009; N-cadherin+ area *P* = 0.0011. Percentage of N-cadherin+ area/endothelial area decreased in Rbpj^iΔEC^ cortex, as compared to controls (right graph in **P**; *P* = 0.0119). *N* = 4 controls and *N* = 4 mutants. **(Q)** RT-qPCR analysis of transcript expression from isolated P14 brain pericytes showed decreased expression of *Pdgfrβ* (*p* = 0.0360), *N-cadherin* (*P* = 0.0084) and *CD146* (*P* = 0.0002) in Rbpj^iΔEC^ mutants as compared to controls. **(R)** Western blot analysis of protein expression from isolated brain pericytes and ECs showed decreased expression of Pdgfrβ (*P* = 0.0349) and N-cadherin (*P* = 0.0487) in Rbpj^iΔEC^ pericytes [quantified in **(S,T)**, respectively; *N* = 3 controls and *N* = 3 mutants] and increased expression of N-cadherin (*P* = 0.0143) in Rbpj^iΔEC^ ECs [quantified in **(V)**; *N* = 4 controls and *N* = 4 mutants]. **(U)** RT-qPCR analysis of transcript expression from isolated P14 brain ECs showed increased expression of *N-cadherin* (*P* = 0.0216) in Rbpj^iΔEC^ ECs. *N* = 3 controls and *N* = 3 mutants. *P < 0.05; ^**^P < 0.01; ^***^P < 0.001.

Based on previous reports of Notch dependent Pdgfrβ expression in pericytes, we next hypothesized that endothelial Rbpj deficiency from birth would lead to decreased Pdgfrβ expression in pericytes by P14. We first examined Pdgfrβ+ pericyte area to determine whether Rbpj^iΔEC^ pericyte expansion included predominately Pdgfrβ+ or Pdgfrβ- pericytes. We measured Pdgfrβ+ and mGFP+ areas in P14 cerebellum and cortex. As expected, and consistent with previous data, mGFP+ endothelial area was increased in both P14 cerebellum and cortex (cerebellum Figures 5A–C, left graph in D; cortex Figures 5E–G, left graph in H). In P14 cerebellum and cortex, we found slight but significantly increased Pdgfrβ+ area in mutants, as compared to controls (Figures 5A–H). By contrast to our CD13+ and desmin+ area data, the percentage of Pdgfrβ+ area/endothelial area was significantly decreased in P14 Rbpj^iΔEC^ cerebellum and cortex (right graphs in Figures 5D,H). These data suggest that while total pericyte area increased in Rbpj^iΔEC^ brains, a significant number of pericytes expressed Pdgfrβ abnormally. To determine whether endothelial deletion of Rbpj affected pericyte *Pdgfrβ* expression at the transcript and/or protein level, we isolated pericytes from P14 control and Rbpj^iΔEC^ brains for transcript and Western blot analyses. As compared to controls, Rbpj^iΔEC^ brain pericytes showed decreased expression of *Pdgfrβ* transcript (Figure 5Q) and protein (Figures 5R,S) by P14, indicating that endothelial Rbpj regulates expression of *Pdgfrβ* in pericytes in the early postnatal brain.

### Endothelial Rbpj deficiency led to decreased N-cadherin and *CD146* expression in pericytes, but increased N-cadherin expression in endothelial cells

We next tested whether endothelial deletion of Rbpj affected expression of other factors involved in EC-pericyte communication—specifically, molecules predicted to be regulated by (N-cadherin) or molecules predicted to be regulators of (CD146) Pdgf-B/Pdgfrβ signaling. We immunostained against N-cadherin, a molecule expressed by ECs and pericytes and involved in direct cell-cell association. We measured N-cadherin+ and mGFP+ areas in P14 cerebellum and cortex. As expected, and consistent with previous data, mGFP+ endothelial area was increased in both P14 cerebellum and cortex (cerebellum Figures 5I–K, left graph in L; cortex Figures 5M–O, left graph in P). Total N-cadherin+ area was significantly increased in both cerebellum (Figure 5L, middle graph) and cortex (Figure 5P, middle graph). The percentage of N-cadherin+ area/endothelial area was significantly increased in P14 Rbpj^iΔEC^ cerebellum and cortex (right graphs in Figures 5L,P); however, it must be noted that N-cadherin expression was not exclusive to pericytes, so the percentage did not represent pericyte/endothelial coverage exclusively. To determine whether endothelial deletion of Rbpj affected EC and/or pericyte expression of *N-cadherin* at the transcript and/or protein level, we isolated ECs and pericytes from P14 control and Rbpj^iΔEC^ brains. As compared to controls, P14 Rbpj^iΔEC^ brain pericytes showed decreased expression of *N-cadherin* transcript (Figure 5Q) and protein (Figures 5R,T). By contrast, P14 Rbpj^iΔEC^ brain ECs showed increased expression of *N-cadherin* transcript (Figure 5U) and protein (Figures 5R,V), indicating that endothelial Rbpj regulates expression of *N-cadherin* differentially in pericytes and ECs in the early postnatal brain.

CD146 is expressed dynamically by ECs and pericytes in the developing brain, where it has been shown to promote pericyte association with ECs, in part by regulating Pdgf-B/Pdgfrβ signaling. In brain microvessels, CD146 is expressed primarily by pericytes, where it has been shown to act as a co-receptor for Pdgfrβ (Chen et al., 2017). To test whether expression of *CD146* was affected by endothelial Rbpj deficiency, we analyzed *CD146* expression in pericytes isolated from P14 control and Rbpj^iΔEC^ brains. We found decreased expression of *CD146* by Rbpj^iΔEC^ brain pericytes, as compared to controls (Figure 5Q), suggesting that endothelial Rbpj regulates expression of *CD146*. Together, our data suggest that Rbpj is required in early postnatal endothelium, to regulate expression of molecules involved in EC-pericyte communication and association.

## Discussion

### Endothelial Rbpj regulates pericyte bed area and maintains pericyte morphology in the early postnatal central nervous system

**FIGURE 6:**
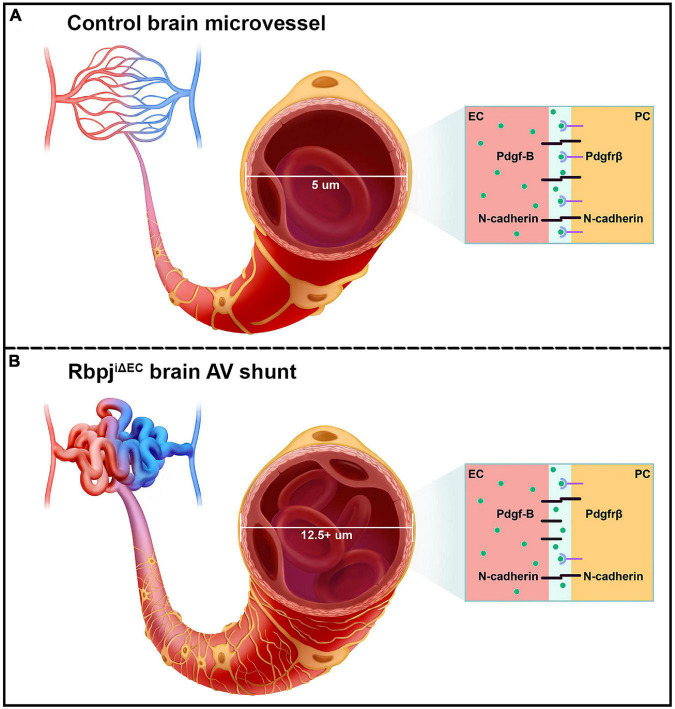
Schematic of the pathological consequences to brain pericytes, following endothelial deletion of Rbpj. **(A)** Top cartoon illustrates healthy brain microvessels. In controls, ∼5 μm capillaries are enwrapped by pericyte processes (gold). Capillary ECs and pericytes are tightly apposed and share a basement membrane. Box represents an adjacent EC and pericyte (PC), which remain tightly associated via Pdgf-B/Pdgfrβ signaling and downstream N-cadherin expression. **(B)** Bottom cartoon illustrates Rbpj^iΔEC^ AV connections. Vessel diameter is enlarged (12.5+ μm), and pericytes pathologically expand to keep pace with the increased endothelium. Box shows effects on EC-pericyte communication, following endothelial deletion of Rbpj: pericytes down-regulate Pdgfrβ and N-cadherin, while ECs up-regulate N-cadherin.

Our data indicate that endothelial Rbpj is required to prevent pathological pericyte expansion in the early postnatal brain vasculature (Figures 6A,B). Consistent with our data, previous studies have shown increased pericyte area or coverage in abnormal CNS or non-CNS vascular beds. Loss of *Foxf2* from neuronal progenitor cells leads to a twofold increase in pericyte coverage of forebrain cortical microvessels in late-gestation mouse embryos (Reyahi et al., 2015), suggesting genetic regulation to prevent pericyte expansion in the developing CNS. Depletion of astrocytes in perinatal mouse brain leads to increased cortical vessel and luminal diameter, with increased desmin+ pericyte expression, suggesting that newly born astrocytes influence the early postnatal brain microvessels and pericytes (Ma et al., 2012). Outside of the CNS vasculature, increased pericyte coverage has been reported in bone marrow samples from myelofibrosis patients (Zetterberg et al., 2007), in PDGF-BB induced non-small cell lung xenograft tumors (Song et al., 2009), in an *in vitro* pulmonary hypertension model (Khoury and Langleben, 1996), and in mice and guinea pig cochlear capillaries following auditory trauma (Shi, 2009). Our pericyte expansion data are consistent with these previous findings, which demonstrate increased pericyte area and/or coverage, in the context of microvessel abnormalities.

In the early postnatal brain, endothelial Rbpj likely plays a role in maintaining pericyte morphology and regulating the number of pericytes per vessel length. Our EdU incorporation data indicate that pericyte proliferation was not increased in Rbpj^iΔEC^ brain vasculature, yet total pericyte area and pericyte number per vessel length were increased. Based on our whole-mount observations of desmin+ pericyte processes enwrapping abnormal AV connections in Rbpj^iΔEC^ brain, it is likely that pericytes abnormally extend processes, in response to endothelial Rbpj deficiency and pathological endothelial expansion. As brain AVM pathology progressed, pericytes on Rbpj^iΔEC^ AV shunts began to resemble the band-like morphology characteristic of smooth muscle cells. However, previous data showed that α-smooth muscle actin (αSMA, a smooth muscle marker) was not expressed by brain AV shunts in P14 Rbpj^iΔEC^ mice, while αSMA was expressed by arterioles (Nielsen et al., 2014). Since the band-like desmin+ pericytes spanned the entire length of the AV shunt in Figure 4, it is not likely that these are smooth muscle cells, but rather pericytes that have altered band-like morphology.

What then accounts for the increased number of pericytes on AV shunts, as compared to control capillaries? As brain pericytes are capable of microvascular migration (Dore-Duffy et al., 2000) and of cellular trans-differentiation (Dore-Duffy et al., 2006; Bonkowski et al., 2011) within the CNS, it is possible that affected pericytes migrate from healthy vessel segments to abnormal AV connections and/or initiate differentiation into another neural cell type, yet retain pericyte marker expression and position adjacent to blood vessels. Because mesenchymal cells, bone marrow cells, and ECs are capable of trans-differentiating into pericytes—either *in vitro* or under pathological conditions (DeRuiter et al., 1997; Rajantie et al., 2004; Kokovay et al., 2006)—it is possible that these origins contribute to increased pericyte number per vessel length in Rbpj^iΔEC^ brains. Another possibility is that the nature of brain AV shunt formation in Rbpj^iΔEC^ mice influences pericyte number per vessel length. For example, in healthy early postnatal brain vasculature, vascular remodeling is required to form a functionally mature vascular bed (Wang et al., 1992; Wälchli et al., 2015). At P0–P5, mouse neurovasculature resembles a primitive vascular plexus (similar to the yolk sac vasculature) in which arteries and veins can be identified, but in which AV connections have not yet refined to capillary-diameter vessels (Wang et al., 1992; Nielsen et al., 2014). By P14, capillary beds can be seen joining arterial to venous vessels (Nielsen et al., 2014). While pericyte dynamics during this postnatal morphogenesis have not been studied, it is possible that impaired vascular remodeling affects pericyte number in Rbpj^iΔEC^ neonatal brain tissue. For example, AV connection remodeling may involve EC rearrangement by which vessels narrow and “stretch out,” and this may affect pericyte number per given vessel length. Thus, endothelial Rbpj may be required in the early postnatal brain to maintain healthy pericyte number and morphology.

### Endothelial Rbpj regulates central nervous system pericyte bed area in a regionally and temporally regulated manner

Our findings show that abnormal pericyte expansion began in cerebellum and retina by P10 and in frontal cortex by P14, and pericyte area expansion continued until P21 in both brain regions; thus, endothelial Rbpj influences pericytes differentially as postnatal brain morphogenesis proceeds. Pericyte expansion paralleled endothelial expansion at all timepoints, in both cerebellum and cortex, indicating that pericyte area keeps pace with pathological endothelial area, following endothelial deletion of Rbpj. Cerebellum pericytes were preferentially affected in mutants, pericyte expansion began earliest (P10) and was most severe by P14 in the cerebellum (Table 1). These findings are consistent with significant abnormalities to cerebellum tissue itself, following endothelial deletion of Rbpj (Chapman et al., 2022). During the early postnatal period, endothelial Rbpj is critical for cerebellar vascular development and proper tissue lobulation and growth (Chapman et al., 2022). Our findings show that endothelial Rbpj is also critical for pericyte maintenance during this period of cerebellar morphogenesis and outward growth. By contrast, pericyte area was not affected in the brain stem at any timepoint analyzed, suggesting that endothelial Rbpj regulates pericyte expansion in a regionally restricted manner. Consistent with this, (1) endothelial area in brain stem was not affected, following endothelial deletion of Rbpj in postnatal brain (Nielsen et al., 2014), and (2) brain stem AVM in human patients are rare, comprising 2–6% of all brain AVM (Chen et al., 2021) and often presenting superficially in the meninges rather than deep within brain parenchyma (Han et al., 2015). It is not known whether or how the brain stem is physiologically refractory to and/or protected from brain AVM. Together, our data demonstrate spatial and temporal regulation of CNS pericyte area by endothelial Rbpj.

### Endothelial Rbpj regulates expression of factors that promote brain pericyte recruitment and association with endothelial cells

Decreased expression of *Pdgfrβ*, *N-cadherin*, and *CD146* by Rbpj^iΔEC^ brain pericytes suggests that intercellular communication between ECs and pericytes was affected. For example, Pdgf-B/Pdgfrβ signaling between ECs and pericytes is critical for recruiting pericytes to microvessels and maintaining the cells’ close juxtaposition (Armulik et al., 2010; Daneman et al., 2010; Chen et al., 2017; Nikolakopoulou et al., 2017). Despite the expanded pericyte area in Rbpj^iΔEC^ neurovasculature, Pdgf-B/Pdgfrβ signaling was likely affected and pericyte association with microvessels may have been impaired. Consistent with previous studies (Jin et al., 2008; Li et al., 2011; Yao et al., 2011), our data suggest a role for endothelial Rbpj/Notch signaling to regulate pericyte expression of *Pdgfr*β. Interestingly, our results showed that while pericytes decreased *N-cadherin* expression, following endothelial deletion of Rbpj, ECs increased *N-cadherin* expression. This differential regulation of *N-cadherin* by endothelial Rbpj may be an attempt by ECs to compensate for decreased Pdgf-B/Pdgfrβ signaling and N-cadherin expression and thereby restore vessel stability, in the context of Rbpj^iΔEC^ microvessel and pericyte abnormalities. Collectively, our data suggest that endothelial Rbpj regulates Pdgf-B/Pdgfrβ signaling, including expression of genes encoding the Pdgfrβ co-receptor CD146 and downstream target N-cadherin (Figures 6A,B).

As proper pericyte coverage of microvessels is involved in regulating vascular permeability, it is possible that the pathologically expanded pericyte bed in Rbpj^iΔEC^ brain may affect pericyte function and vessel stability. However, given the abnormalities to ECs, following endothelial deletion of Rbpj, it would be difficult to tease apart whether abnormal function of pericytes and/or ECs might contribute to altered vessel permeability. By P14, Rbpj^iΔEC^ brains show signs of intracerebral hemorrhage (Nielsen et al., 2014); however, the cellular mechanism by which blood leaks from Rbpj^iΔEC^ vessels remains unknown. One hypothesis raised by our data is that brain pericytes expand in concert with Rbpj^iΔEC^ vessels, as an attempt to stabilize mutant microvessels.

Recent evidence suggests there is a variety of pericyte subtypes that differ in morphology, molecular signature, and vascular function (Hartmann et al., 2015; Sweeney et al., 2016; Geranmayeh et al., 2019; Lendahl et al., 2019; Uemura et al., 2020; Bennett and Kim, 2021; Nadeem et al., 2022); therefore, the different pericyte populations may be differentially affected by Rbpj mediated brain AVM. We observed differences in pericyte area measurements, based on the which pericyte marker was used for analyses. While this alone underscores the importance of using multiple pericyte markers, this may also provide insight regarding how—and perhaps which—pericytes were pathologically expanded. CD13 is a metalloprotease and considered a selective surface marker for pericytes (Crouch and Doetsch, 2018), while desmin is present in intermediate filaments commonly found in pericyte processes (Armulik et al., 2011). As such, thin desmin+ processes may have been less detectable in mid-sagittal area measurements but gave insight about morphological changes to abnormal pericytes in Rbpj^iΔEC^ brain.

### Pericyte reduction or loss from brain microvessels is not associated with Rbpj mediated brain arteriovenous malformation

To our knowledge, this report is the first to demonstrate pathological pericyte expansion in a mouse model of brain AVM. Our data contrast findings from other genetic models of CNS AVMs and from human brain AVM tissue and thus emphasize the heterogeneity among AVM pathologies and mechanisms. For example, reduced pericyte number and coverage of endothelium was reported in sporadic brain AVM samples from human patients (Winkler et al., 2018); however, these correlative studies could not reveal whether pericyte reduction was a cause or consequence of human brain AVM. Using mouse models of TGFβ related brain AVM, studies have similarly shown reduced pericyte coverage of brain microvessels (Chen et al., 2013; Tual-Chalot et al., 2014). In a genetic model of retina AVM, endothelial Smad4 deficiency led to decreased pericytes associated with retinal vessels (Crist et al., 2018). Two recent studies genetically deleted Rbpj from pericytes, in the early postnatal period, and found CNS AVM with pericyte deficiency (Diéguez-Hurtado et al., 2019; Nadeem et al., 2020). Further, Nadeem et al. (2020) showed that pericyte deficiency preceded vessel enlargement, suggesting that intact pericytes prevent AV shunting in retina vasculature. Differences in pericyte coverage among AVM models may reflect different mechanisms in AVM pathogenesis. For example, some AVMs are triggered by increased EC proliferation, serving to populate the increasing-diameter vessel growth. Such angiogenic AVMs are associated with reduced pericyte coverage, possibly as a mechanism to accommodate proliferative ECs and growth. By contrast, some AVMs, including Rbpj^iΔEC^ mediated brain AVMs, develop without increased EC proliferation (Murphy et al., 2014; Nielsen et al., 2014)—perhaps non-angiogenic ECs on microvessels elicit different pericyte responses during AVM pathogenesis. Despite the contrasting data for pericytes and CNS AVMs, our findings show that pericyte deficiency is not required for brain AVM formation. Thus, our study underscores the importance of probing mechanisms of genetically distinct brain AVM and of developing specific and appropriate therapies.

A question remains regarding the functional implications of pericyte abnormalities during brain AVM. In models of pericyte reduction during brain AVM, one working hypothesis is that the loss of pericytes loosens contractile constraints on microvessels and permits vessel dilation (Nadeem et al., 2020). Following even a slight increase in vessel diameter, increased blood flow is directed toward that path of least resistance and triggers continued vessel dilation, resulting in AV shunting. Loss of pericytes in brain AVM has also been suggested to disrupt vessel stability, which may be related to intracranial hemorrhages associated with brain AVM tissue (Winkler et al., 2018). Data from our Rbpj mediated brain AVM model showed that pericyte expansion was accompanied by abnormalities to pericyte morphology and disruptions to EC-pericyte communication. Thus, these abnormal pericytes may also contribute to microvascular instability. Because endothelial Rbpj deletion leads to endothelial, pericyte, cerebellar, and motor behavioral abnormalities (Nielsen et al., 2014; Chapman et al., 2022), a future challenge will be to tease apart which brain AVM pathologies are a consequence of endothelial vs. pericyte vs. tissue morphogenetic impairments, or of some combination thereof.

## Data availability statement

The original contributions presented in this study are included in the article/Supplementary material, further inquiries can be directed to the corresponding author.

## Ethics statement

This animal study was reviewed and approved by the Ohio University IACUC, Animal Protocol # 16-H-024.

## Author contributions

SS, SN, SK, and CN conceptualized and designed the experiments. SS, SN, SK, SA, and CN performed the experiments and analyzed the data. CN wrote the manuscript. All authors contributed to the article and approved the submitted version.
