# Supplementary material for: Pathological pericyte expansion and impaired endothelial cell-pericyte communication in endothelial Rbpj deficient brain arteriovenous malformation
Source: Front Hum Neurosci. 2022 Sep 6;16:974033. doi: 10.3389/fnhum.2022.974033 (PMC9485665; doi:10.3389/fnhum.2022.974033)
Supplement: Supplementary file 1 [file Data_Sheet_1.pdf]

Supplementary Material

Supplementary Figures and Tables

Supplementary Figure 1

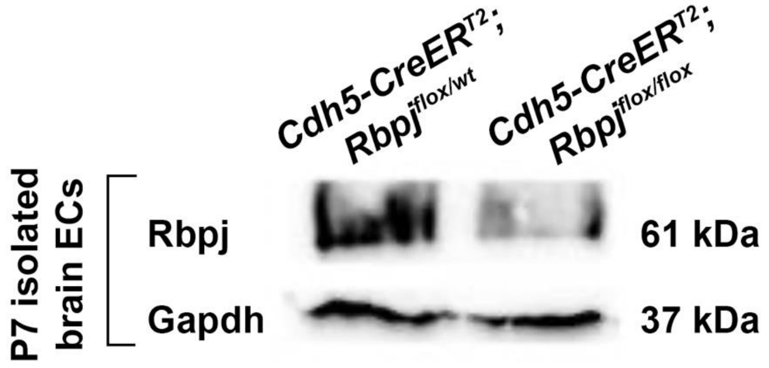

Supplementary Figure 1. Rbpj protein was effectively reduced in isolated brain ECs from P7 Rbpj$^{i\Delta EC}$ mutants, as compared to controls. Western blot analysis showed that Rbpj protein (61 kDa) was present in brain ECs isolated from P7 control mice. Rbpj protein was absent from P7 mutant brain ECs. Gapdh loading control (37 kDa) was expressed by both control and mutant brain ECs.

Supplementary Figure 2

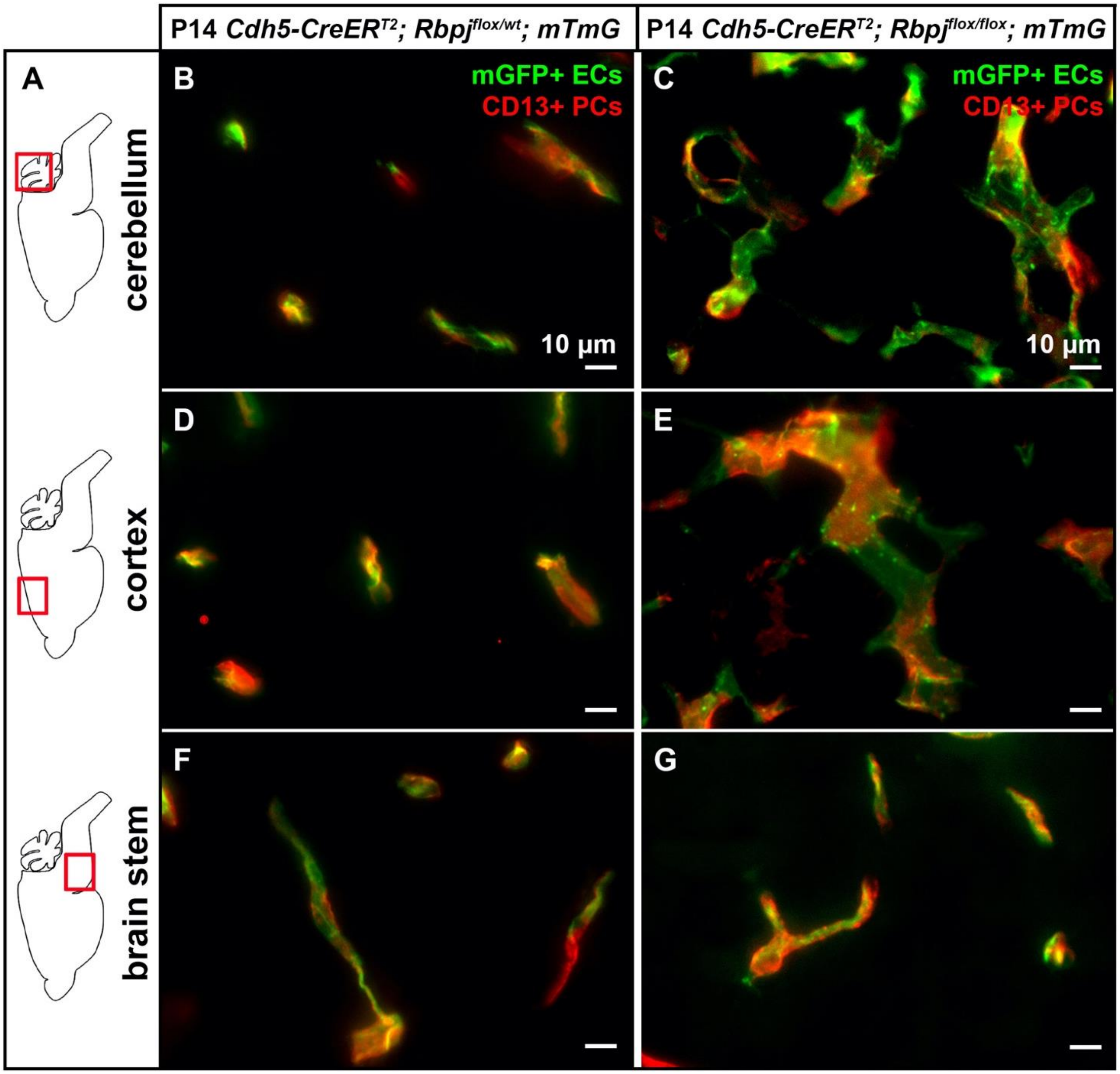

Supplementary Figure 2. Higher magnification images of P14 CD13+ pericytes and mGFP+ microvessels. In all tissue panels, CD13+ pericytes (red) and mGFP+ ECs (green) were imaged from P14 control and $Rbpj^{i\Delta EC}$ brain tissue. (A) Schematics indicate regions of mid-sagittal brain sections imaged. CD13+ pericytes were closely juxtaposed to normal (control) and abnormally enlarged ($Rbpj^{i\Delta EC}$) mGFP+ microvessels in cerebellum (B-C) and frontal cortex (D-E) and to normal caliber vessels in control and $Rbpj^{i\Delta EC}$ brain stem (F-G).

Supplementary Figure 3

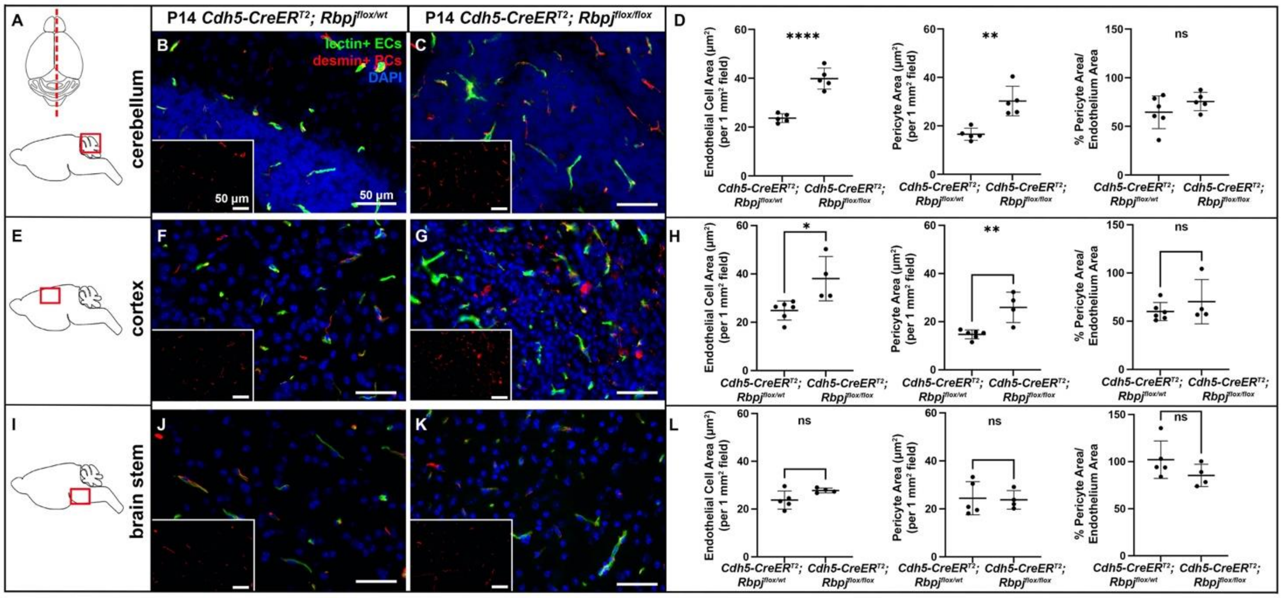

Supplementary Figure 3. Desmin-positive cortex and cerebellum pericyte area expanded and kept pace with expanded endothelium at P14, following endothelial deletion of Rbpj. In all tissue panels, desmin+ pericytes (PCs) (red), mGFP+ ECs (green), DAPI+ nuclei (blue); insets show desmin+ pericytes only. (A) Upper schematic indicates mid-sagittal plane of section through brain. Lower schematic indicates cerebellum region shown. (B-C) mGFP+ endothelial area and desmin+ pericyte area pathologically expanded in P14 Rbpj$^{i\Delta EC}$ cerebellum, as compared to controls. Quantified in (D): endothelial area P<0.0001; pericyte area P=0.0017. Percentage of pericyte area/endothelial area did not change (right graph in D; P=0.2242. N=6 controls and N=5 mutants. (E) Schematic indicates cortex region shown. (F-G) mGFP+ endothelial area and desmin+ pericyte area pathologically expanded in P14 Rbpj$^{i\Delta EC}$ cortex, as compared to controls. Quantified in (H): endothelial area P=0.0131; pericyte area P=0.0030. Percentage of pericyte area/endothelial area did not change (right graph in H; P=0.3491). N=6 controls and N=4 mutants. (I) Schematic indicates brainstem region shown. (J-K) mGFP+ endothelial area and desmin+ pericyte area did not change in P14 Rbpj$^{i\Delta EC}$ brain stem, as compared to controls. Quantified in (L): endothelial area P=0.0812; pericyte area P=0.8663. Percentage of pericyte area/endothelial area did not change (right graph in L; P=0.1830). N=5 controls and N=4 mutants.

Supplementary Figure 4

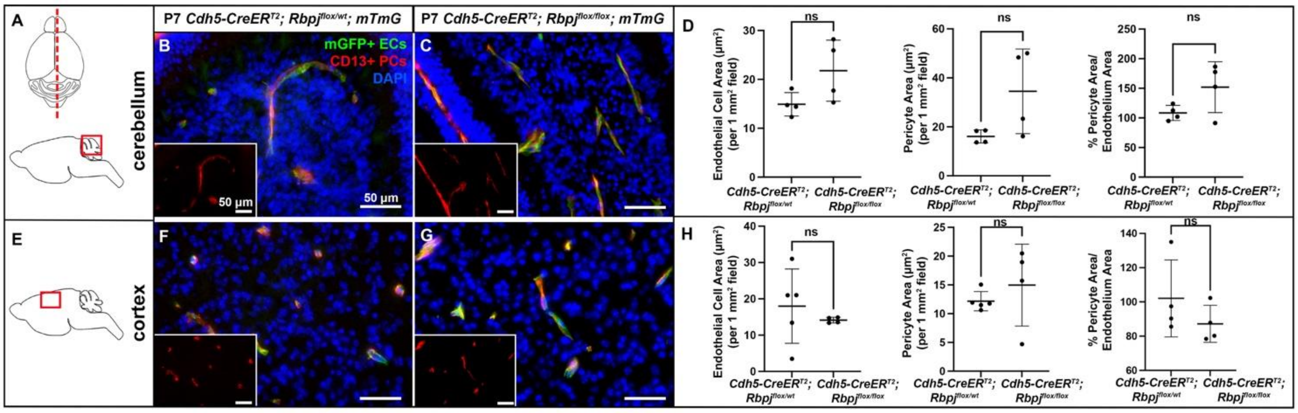

Supplementary Figure 4. CD13-positive pericytes were present on cerebellum and cortex microvessels at P7, following endothelial deletion of Rbpj. In all tissue panels, CD13+ pericytes (PCs) (red), mGFP+ ECs (green), DAPI+ nuclei (blue); insets show CD13+ pericytes only. (A) Upper schematic indicates mid-sagittal plane of section through brain. Lower schematic indicates cerebellum region shown. (B-C) mGFP+ endothelial area and CD13+ pericyte area were not changed in P7 Rbpj$^{i\Delta EC}$ cerebellum, as compared to controls. Quantified in (D): endothelial area P=0.0856; pericyte area P=0.0799. Percentage of pericyte area/endothelial area did not change (right graph in D; P=0.0992. N=4 controls and N=4 mutants. (E) Schematic indicates cortex region shown. (F-G) mGFP+ endothelial area and CD13+ pericyte area were not changed in P7 Rbpj$^{i\Delta EC}$ cortex, as compared to controls. Quantified in (H): endothelial area P=0.4822; pericyte area P=0.4175. Percentage of pericyte area/endothelial area did not change (right graph in H; P=0.2784). N=5 controls and N=4 mutants.

Supplementary Figure 5

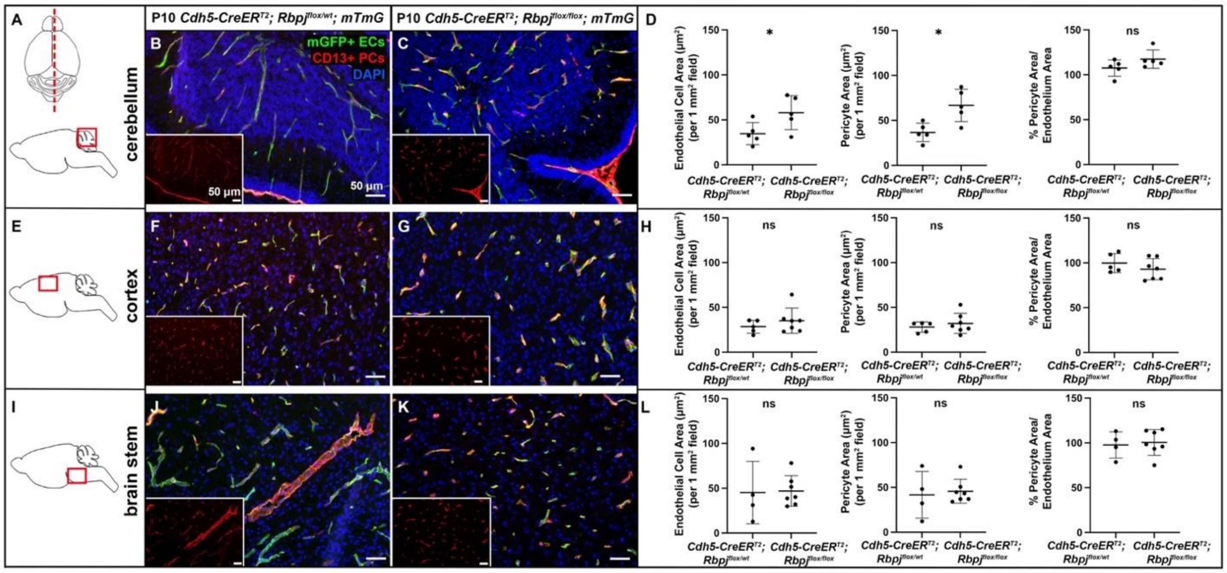

Supplementary Figure 5. CD13-positive cerebellum pericyte area expanded and kept pace with expanded endothelium at P10, following endothelial deletion of Rbpj. In all tissue panels, CD13+ pericytes (PCs) (red), mGFP+ ECs (green), DAPI+ nuclei (blue); insets show CD13+ pericytes only. (A) Upper schematic indicates mid-sagittal plane of section through brain. Lower schematic indicates cerebellum region shown. (B-C) mGFP+ endothelial area and CD13+ pericyte area pathologically expanded in P10 Rbpj$^{i\Delta EC}$ cerebellum, as compared to controls. Quantified in (D): endothelial area P=0.0498; pericyte area P=0.0120. Percentage of pericyte area/endothelial area did not change (right graph in D; P=0.1426. N=5 controls and N=5 mutants. (E) Schematic indicates cortex region shown. (F-G) mGFP+ endothelial area was expanded but CD13+ pericyte area was not changed in P10 Rbpj$^{i\Delta EC}$ cortex, as compared to controls. Quantified in (H): endothelial area P=0.3576; pericyte area P=0.4709. Percentage of pericyte area/endothelial area did not change (right graph in H; P=0.3252). N=5 controls and N=7 mutants. (I) Schematic indicates brainstem region shown. (J-K) mGFP+ endothelial area and CD13+ pericyte area did not change in P10 Rbpj$^{i\Delta EC}$ brain stem, as compared to controls. Quantified in (L): endothelial area P=0.9130; pericyte area P=0.7351. Percentage of pericyte area/endothelial area did not change (right graph in L; P=0.7579). N=4 controls and N=7 mutants.

Supplementary Figure 6

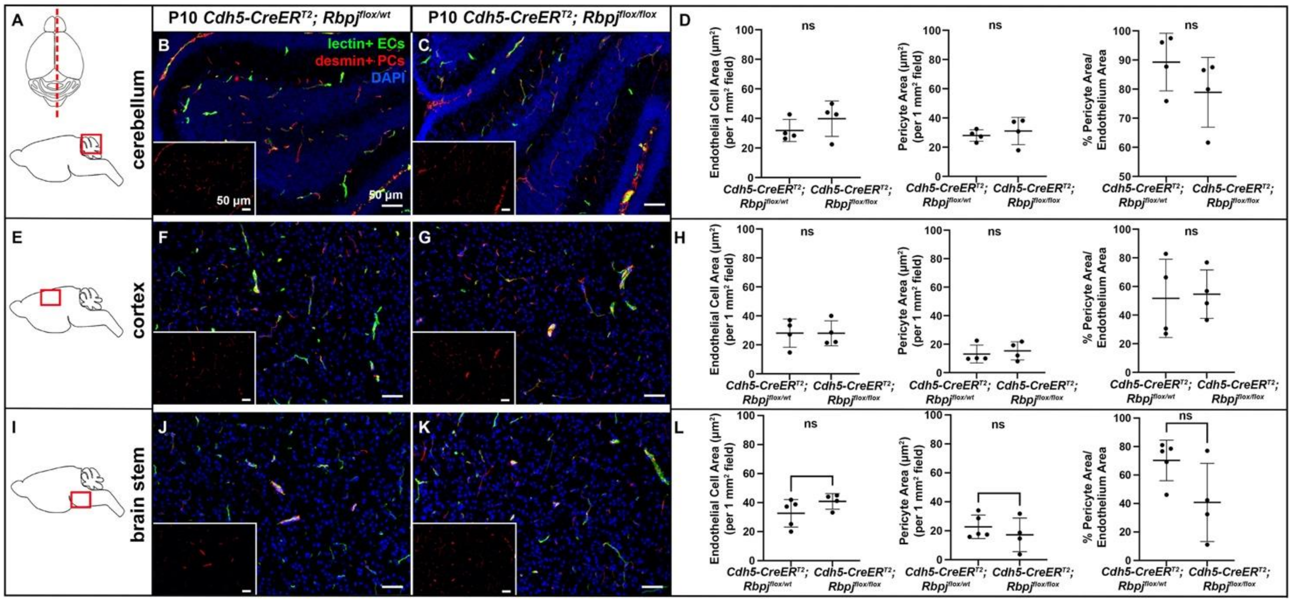

Supplementary Figure 6. Desmin-positive pericyte area showed no change in P10 cortex, cerebellum, and brain stem, following endothelial deletion of Rbpj. In all tissue panels, desmin+ pericytes (PCs) (red), Dylight488-lectin+ ECs (green), DAPI+ nuclei (blue); insets show desmin+ pericytes only. (A) Upper schematic indicates mid-sagittal plane of section through brain. Lower schematic indicates cerebellum region shown. (B-C) lectin+ endothelial area and desmin+ pericyte area did not change in P10 Rbpj$^{i\Delta EC}$ cerebellum, as compared to controls. Quantified in (D): endothelial area P=0.3020; pericyte area P=0.5665. Percentage of pericyte area/endothelial area did not change (right graph in D; P=0.2303. N=4 controls and N=4 mutants. (E) Schematic indicates cortex region shown. (F-G) lectin+ endothelial area and desmin+ pericyte area did not change in P10 Rbpj$^{i\Delta EC}$ cortex, as compared to controls. Quantified in (H): endothelial area P=0.9867; pericyte area P=0.6361. Percentage of pericyte area/endothelial area did not change (right graph in H; P=0.8625). N=4 controls and N=4 mutants. (I) Schematic indicates brainstem region shown. (J-K) lectin+ endothelial area and desmin+ pericyte area did not change in P10 Rbpj$^{i\Delta EC}$ brain stem, as compared to controls. Quantified in (L): endothelial area P=0.1703; pericyte area P=0.4234. Percentage of pericyte area/endothelial area did not change (right graph in L; P=0.0737). N=5 controls and N=4 mutants.

Supplementary Figure 7

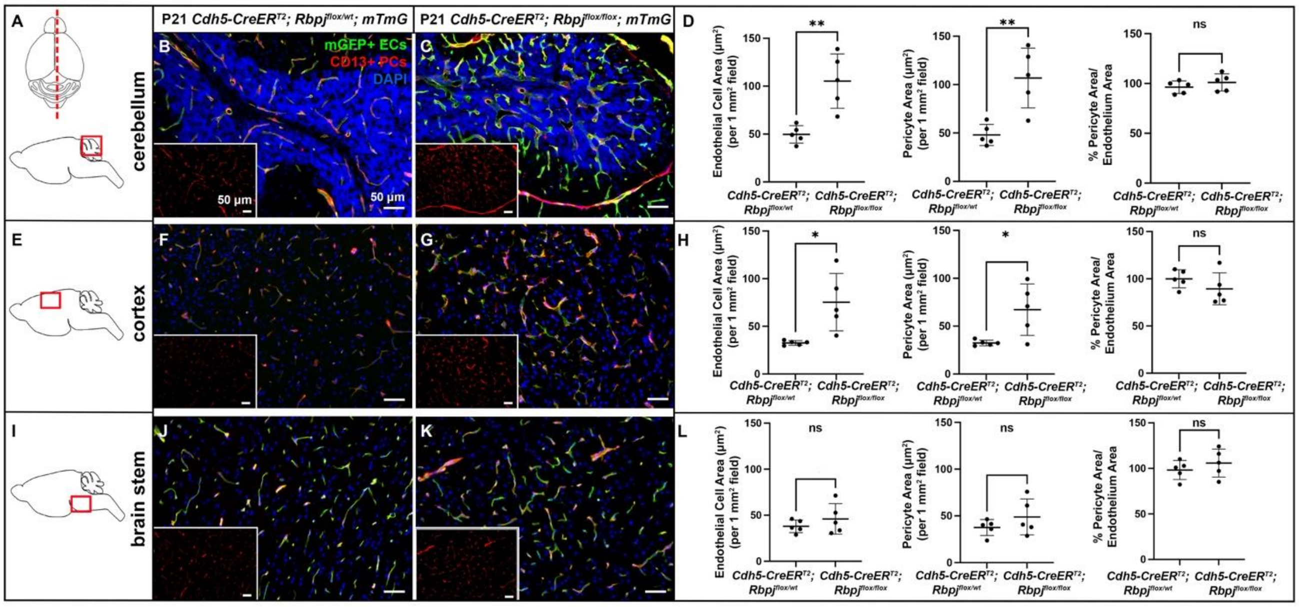

Supplementary Figure 7. CD13-positive cortex and cerebellum pericyte area expanded and kept pace with expanded endothelium at P21, following endothelial deletion of Rbpj. In all tissue panels, CD13+ pericytes (PCs) (red), mGFP+ ECs (green), DAPI+ nuclei (blue); insets show CD13+ pericytes only. (A) Upper schematic indicates mid-sagittal plane of section through brain. Lower schematic indicates cerebellum region shown. (B-C) mGFP+ endothelial area and CD13+ pericyte area pathologically expanded in P21 $Rbpj^{i\Delta EC}$ cerebellum, as compared to controls. Quantified in (D): endothelial area P=0.0031; pericyte area P=0.0038. Percentage of pericyte area/endothelial area did not change (right graph in D; P=0.3428. N=5 controls and N=5 mutants. (E) Schematic indicates cortex region shown. (F-G) mGFP+ endothelial area and CD13+ pericyte area pathologically expanded in P21 $Rbpj^{i\Delta EC}$ cortex, as compared to controls. Quantified in (H): endothelial area P=0.0133; pericyte area P=0.0207. Percentage of pericyte area/endothelial area did not change (right graph in H; P=0.2596). N=5 controls and N=5 mutants. (I) Schematic indicates brainstem region shown. (J-K) mGFP+ endothelial area and CD13+ pericyte area did not change in P21 $Rbpj^{i\Delta EC}$ brain stem, as compared to controls. Quantified in (L): endothelial area P=0.3457; pericyte area P=0.2624. Percentage of pericyte area/endothelial area did not change (right graph in L; P=0.3864). N=5 controls and N=5 mutants.

Supplementary Figure 8

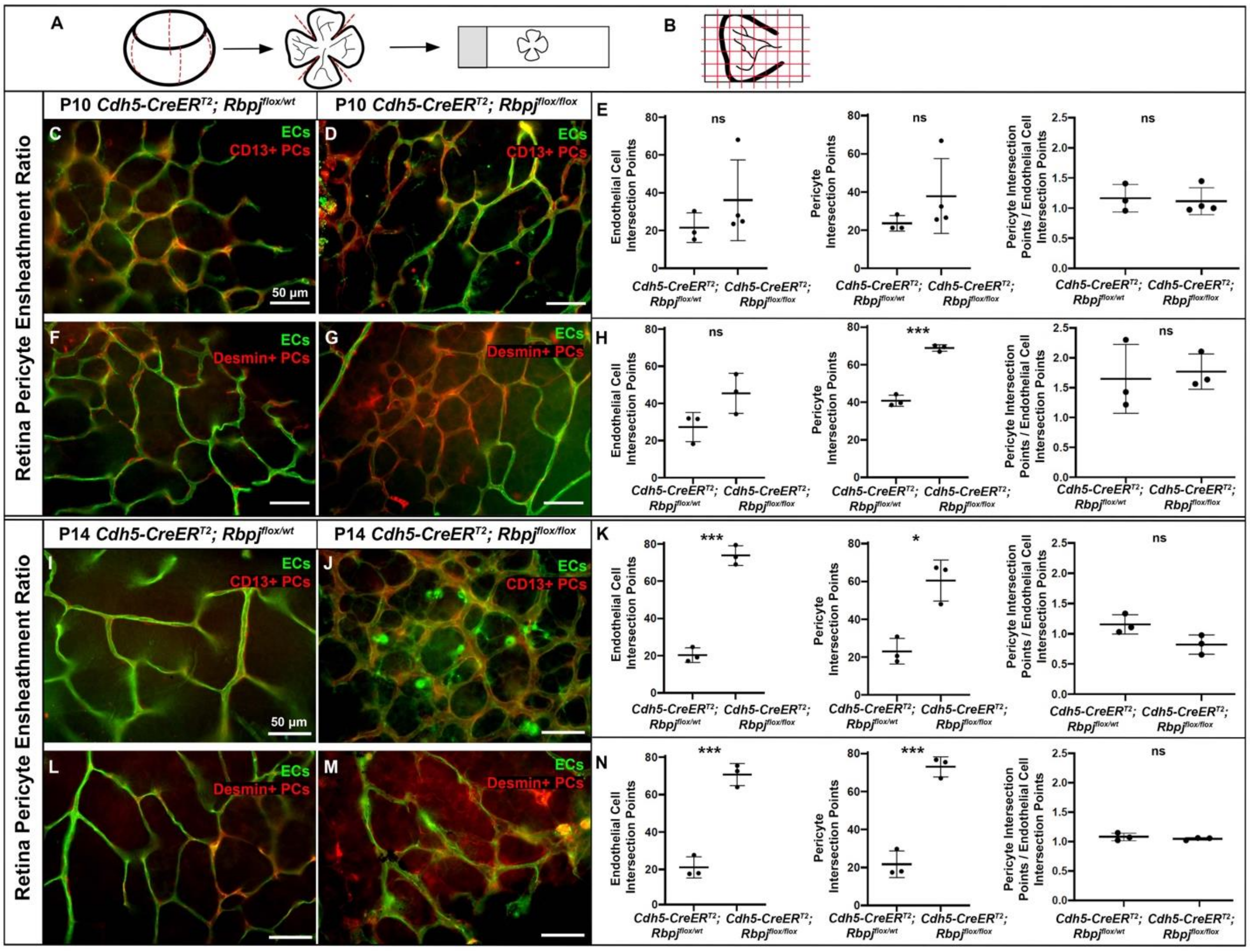

Supplementary Figure 8. Pathological pericyte expansion in the retina vasculature began at P10 and continued through P14, following endothelial deletion of Rbpj. (A) Schematic of mouse retina dissection and preparation for whole mount imaging. (B) Pericyte Ensheathment Ratio (PER) was obtained by pericyte intersection points/endothelial intersection points. Analysis included overlaying a 10 by 10 grid onto immunostained retina images and counting grid intersection points with CD13+ or desmin+ pericytes (PCs) (red) and Dylight488-lectin+ ECs (green). (C-D) CD13+ pericytes were associated with endothelium in control and Rbpj$^{i\Delta EC}$ P10 retina. (E) The numbers of endothelial (P=0.2767) and CD13+ pericyte (P=0.2420) intersection points were not different in P10 Rbpj$^{i\Delta EC}$, as compared to controls (left and middle graphs). Pericyte coverage of endothelium, represented as PER was not different in P10 CD13+ retina (P=0.7870, N=3 controls and N=4 mutants). (F-G) Desmin+ pericytes were associated with endothelium in control and Rbpj$^{i\Delta EC}$ P10 retina. (H) The number of endothelial intersection points (though trending toward increased) was not changed (P=0.0825), while the number of desmin+ pericyte intersection points increased (P=0.0006) in P10 Rbpj$^{i\Delta EC}$, as compared to controls (left and middle graphs). PER was not changed in P10 desmin+ retina (P=0.7685, N=3 controls and N=3 mutants). (I-J) CD13+ pericytes were associated with endothelium in control and Rbpj$^{i\Delta EC}$ P14 retina. (K) The numbers of endothelial (P=0.0002) and CD13+ pericyte (P=0.0111) intersection points were increased in P14 Rbpj$^{i\Delta EC}$, as compared to controls (left and middle graphs). PER was not changed in P14 CD13+ retina (P=0.0621, N=3

controls and N=3 mutants). (L-M) Desmin+ pericytes were associated with endothelium in control and $Rbpj^{i\Delta EC}$ P14 retina. (N) The numbers of endothelial (P=0.0005) and desmin+ pericyte (P=0.0007) intersection points were increased in P14 $Rbpj^{i\Delta EC}$, as compared to controls (left and middle graphs). PER was not changed in P14 desmin+ retina (P=0.4768, N=3 controls and N=3 mutants).

Supplementary Figure 9

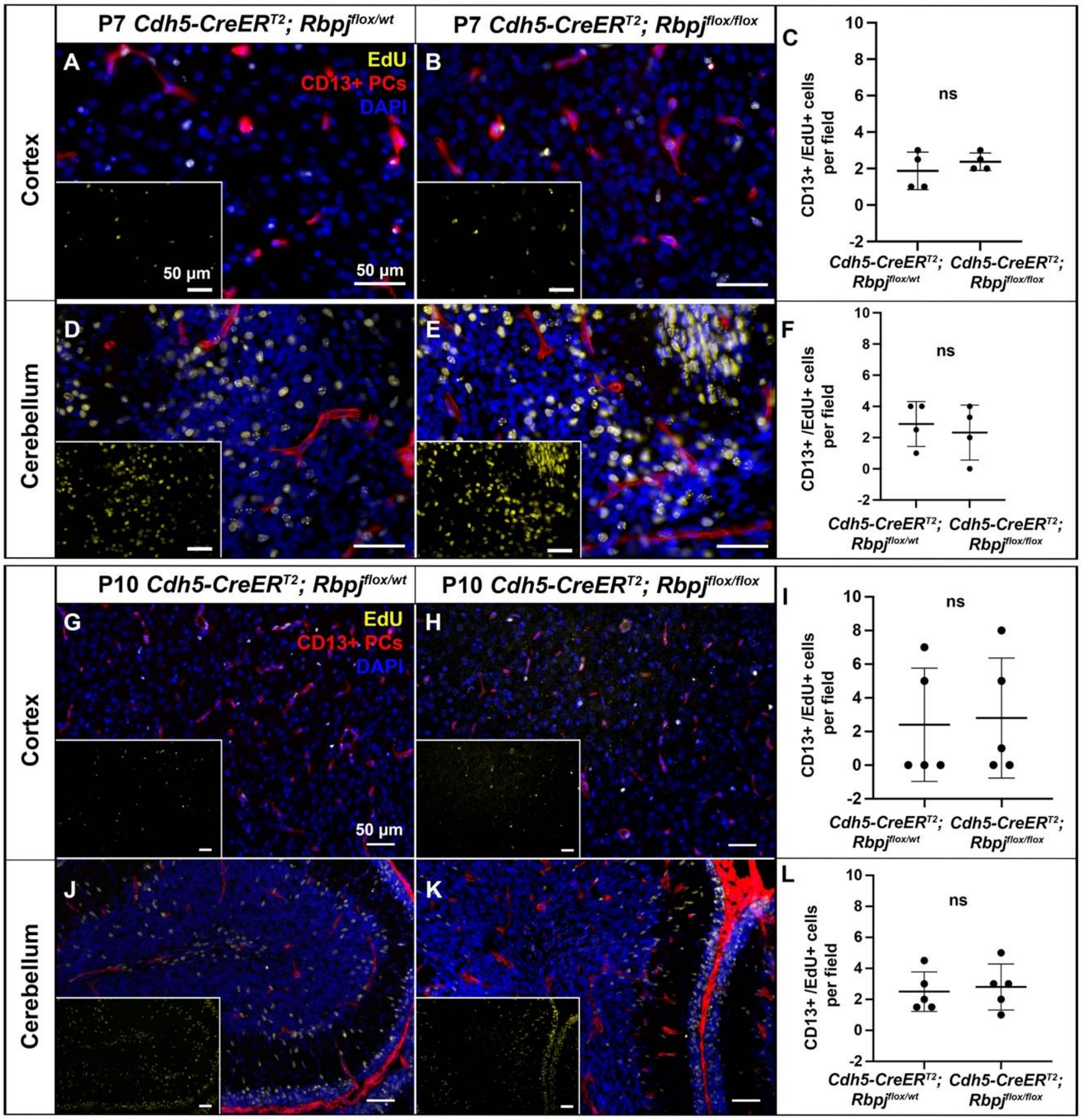

Supplementary Figure 9. EdU incorporation was not changed in cortex and cerebellum pericytes from P5-P7 or from P8-P10, following endothelial deletion of Rbpj. In all tissue panels, CD13+ pericytes (PCs) (red), EdU+ nuclei (yellow), DAPI+ nuclei (blue). (A-B) Mice were administered EdU at P5, P6, P7. Mid-sagittal sections through P7 control and $Rbpj^{i\Delta EC}$ cortex. CD13+ pericytes and CD13+/EdU+ double positive pericytes were counted at P7 harvest. (C) Quantification of CD13+ pericytes per CD13+/EdU+ pericytes showed no significant change (P=0.4260). N=4 controls and N=4 mutants. (D-E) Mid-sagittal sections through P7 control and $Rbpj^{i\Delta EC}$ cerebellum. CD13+ pericytes and CD13+/EdU+ double positive pericytes were counted at P7 harvest. (F) Quantification of CD13+ pericytes per CD13+/EdU+ pericytes showed no significant change (P=0.6513). N=4 controls and N=4 mutants. (G-H) Mice were administered EdU at P8, P9, P10. Mid-sagittal sections through P10 control and $Rbpj^{i\Delta EC}$ cortex. CD13+ pericytes and CD13+/EdU+ double positive pericytes were counted at P10 harvest. (I) Quantification of CD13+

pericytes per CD13+/EdU+ pericytes showed no significant change (P=0.8597). N=5 controls and N=5 mutants. (J-K) Mid-sagittal sections through P10 control and $Rbpj^{i\Delta EC}$ cerebellum. CD13+ pericytes and CD13+/EdU+ double positive pericytes were counted at P10 harvest. (L) Quantification of CD13+ pericytes per CD13+/EdU+ pericytes showed no significant change (P=0.7406). N=5 controls and N=5 mutants.

Supplementary Table 1

Supplementary Table 1. ***Mus musculus*** **primers used for RT-qPCR**

Transcript	Accession number(s)	Variant	Primer sequence	Amplicon size
β-actin	**NM_007393.5**<br>**GI: 930945786**		**(Forward) GTGACGTTGACATCCGTAAAGA**<br>**(Reverse) GCCGGACTCATCGTACTCC**	**132 bp**
CD13	**NM_008486**<br>**XM_006540678**		**(Forward) CCTTCAACCTGGCCAGTGC**<br>**(Reverse) CGTCTTCTCCAGGGCTTGCTCCAG**	**839 bp**
CD146	**NM_001359530**<br>**XM_006510716**	**Variant 2**	**(Forward) AGGACCTTGAGTTTGAGTGG**<br>**(Reverse) CAGTGGTTTGGCTGGAGT**	**480 bp**
Gapdh	**NM_001289726**	**Variant 1**	**(Forward) AGGGTGGACGTCATTGTAGC**<br>**(Reverse) CTGTTGGGGTCTGTCAGGAT**	**145 bp**
N-cadherin	**NM_007664**<br>**XM_904135**<br>**XM_918688**		**(Forward) AGGGTGGACGTCATTGTAGC**<br>**(Reverse) CTGTTGGGGTCTGTCAGGAT**	**134 bp**
Pdgfrβ	**NM_008809**	**Variant 2**	**(Forward) AGCTACATGGCCCCTTATGA**<br>**(Reverse) GGATCCCAAAAGACCAGACA**	**367 bp**
Rplpo	**NM_007475**		**(Forward) TGCTCGACATCACAGAGCAG**<br>**(Reverse) ACGCGCTTGTACCCATTGAT**	**136 bp**
